# Recent Progress in Homogeneous Catalytic Dehydrogenation of Formic Acid

**DOI:** 10.3390/molecules27020455

**Published:** 2022-01-11

**Authors:** Naoya Onishi, Ryoichi Kanega, Hajime Kawanami, Yuichiro Himeda

**Affiliations:** 1 Global Zero Emission Research Center, National Institute of Advanced Industrial Science and Technology, Tsukuba West, 16-1 Onogawa, Tsukuba 305-8569, Ibaraki, Japan; n.onishi@aist.go.jp; 2 Research Institute of Energy Conservation, National Institute of Advanced Industrial Science and Technology, Tsukuba 305-8565, Ibaraki, Japan; r-kanega@aist.go.jp; 3 Interdisciplinary Research Center for Catalytic Chemistry, National Institute of Advanced Industrial Science and Technology, Central 5, 1-1-1 Higashi, Tsukuba 305-8565, Ibaraki, Japan; h-kawanami@aist.go.jp

**Keywords:** formic acid, homogenous catalysts, hydrogen production

## Abstract

Recently, there has been a strong demand for technologies that use hydrogen as an energy carrier, instead of fossil fuels. Hence, new and effective hydrogen storage technologies are attracting increasing attention. Formic acid (FA) is considered an effective liquid chemical for hydrogen storage because it is easier to handle than solid or gaseous materials. This review presents recent advances in research into the development of homogeneous catalysts, primarily focusing on hydrogen generation by FA dehydrogenation. Notably, this review will aid in the development of useful catalysts, thereby accelerating the transition to a hydrogen-based society.

## 1. Introduction

Technological development aimed at building a society that uses hydrogen, which can be used with low environmental load and high efficiency, as an energy medium is urgently required. However, because hydrogen gas is flammable, safety issues involving its storage and transportation limit its use as a fuel [1]. In particular, the research and development of hydrogen carriers for efficiently storing and transporting hydrogen with a low volumetric energy density has become active. In this regard, ammonia [2], ammonia borane [3], and formic acid (FA) [4] have recently been gaining considerable attention. In addition, to make widespread use of hydrogen energy, it is necessary to reduce the cost of supplying hydrogen at hydrogen stations.

Researchers have focused on a hydrogen storage system using FA as a hydrogen carrier. FA is a liquid, containing 4.4 wt% hydrogen, and has slight toxicity to the human body and environment. Another advantage is that carbon dioxide, which is inexpensive and present in large quantities, is used as a raw material. Furthermore, the free energy required for mutual conversion with carbon dioxide in an aqueous solution is −4 kJ/mol, significantly smaller than that of other hydrogen carriers. Therefore, the energy loss required for substance conversion, associated with hydrogen storage and release, can be significantly reduced when FA is used as a hydrogen carrier [5]. In addition, unlike the reactions of other hydrogen carriers, the FA dehydrogenation (FADH) proceeds, even under pressurized conditions; hence, it is possible to supply compressed hydrogen gas. A high-performance homogeneous catalyst is required to utilize FA, which exhibits such excellent properties, as a hydrogen carrier.

In FADH, one possible side reaction (Equation (1) and dehydration) can occur. Generally, CO contaminates fuel cells; thus, a highly active and selective catalyst for FADH (Equation (2)) is strongly desired. Various heterogeneous catalysts for FADH have been studied. However, the activation of these catalysts, such as Pd-based catalysts, usually requires a high temperature, resulting in CO contamination. CO is a well-known poison to the catalyst in the proton exchange membrane (PEM) fuel cells [6]. In addition, these catalysts have low durability and are difficult to perform for long periods of time. Currently, homogeneous catalysts, such as FADH catalysts, are more advantageous than heterogeneous catalysts because they cause less CO contamination and perform at a lower reaction temperature. Coffey performed pioneering work using various homogenous catalysts for FADH [7]. A series of Pt [8], Ru [9], and Rh [10] complexes were evaluated, and the iridium complex IrH_2_Cl(PPh_3_)_3_ gave the highest TOF (1187 h^−1^). Although Pt and Rh complexes were tested for FADH, their catalytic activities and durabilities were low. However, in 2008, a selective FADH, using an effective catalyst, was reported under mild conditions, and the development of homogeneous catalysts became active. This paper summarizes the homogeneous catalysts for FADH and consists of two sections with precious and non-precious metal catalysts.

HCOOH → H_2_O + CO (1)

HCOOH → H_2_ + CO_2_ (2)

## 2. Precious Metal Catalysts for FADH

**Figure 1:**
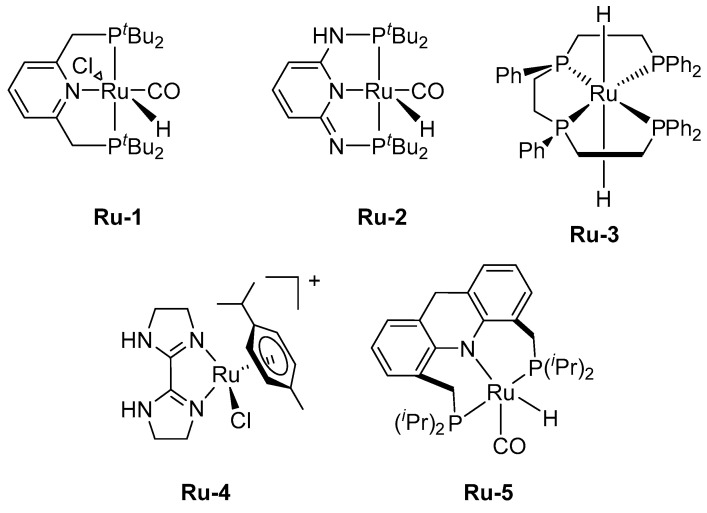
Ru catalysts for FADH.

In 2008, Beller et al. reported a selective FADH, catalyzed by commercially available ruthenium precursors, including [RuCl_2_(PPh_3_)_3_], [RuCl_2_(p-cymene)]_2_, RuCl_2_(PPh_3_)_3_, [RuCl_3_(benzene)]_2_, and RuBr_3_·× H_2_O with triphenylphosphine-type ligands in FA/NEt_3_ azeotropic solutions [11]. The generated gases contained H_2_/CO_2_, without any CO contamination. Additionally, by connecting the reactor to a fuel cell, the authors provided evidence that the hydrogen produced under these conditions could be used to generate electricity directly, without removing CO. No fuel cell catalyst poisoning was observed when all the trace amine vapors were removed from the hydrogen with an activated carbon filter. This process uses the [RuCl_3_(benzene)]_2_ and *N*,*N*-dimethyl-*n*-hexylamine as an optimized catalytic system. Further, 1,2-bis(diphenylphosphino)ethane is used in the setup for continuous hydrogen production. Simultaneous to the discovery by Beller, Laurenczy et al. also reported an effective FADH catalyst. Ru precursor, [Ru(H_2_O)_6_]^2+^ and two equivalents of trisulfonated triphenylphosphine could catalyze FADH in 90 h to generate H_2_/CO_2_ with TON of up to 4000 [12]. Various precious metal catalysts have been developed since the reports of Beller and Laurenczy (Figure 1, Table 1).

**Figure 2:**
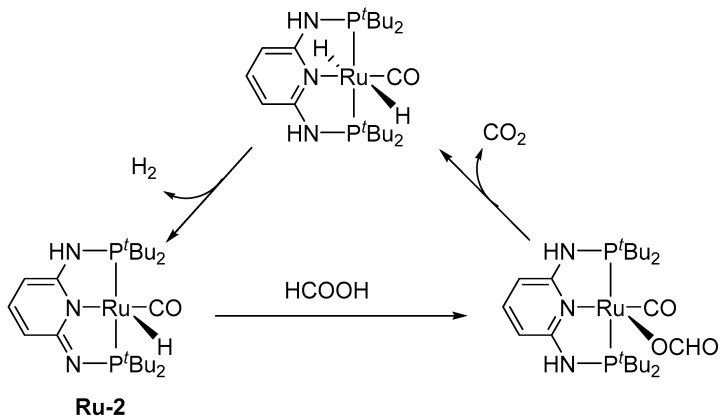
Proposed mechanism for FADH catalyzed by **Ru-2**.

Pincer ligands are used in various catalysts because they provide high thermal stability [13]. Various pincer catalysts have also been developed for FADH. In 2014, Pidko et al. reported a well-defined Ru complex (**Ru-1**), with a PNP-pincer ligand for FADH, with a TOF of up to 257,000 h^−1^ and TON of 706,500 (DMF/NEt_3_, at 90 °C), without a CO byproduct [13]. Because the catalyst performed the hydrogenation of CO_2_ to formate with high activity, the researchers achieved 10 consecutive H_2_ storage/generation cycles in a DMF/DBU solvent mixture over 1 week. Huang and Zheng et al. studied a Ru complex (**Ru-2**) with a PNP-pincer ligand, a dearomatized pyridine moiety with an imine arm [14]. Using this catalyst for FADH, a TON of 95,000 was obtained under mild conditions without any additive (50 °C, in DMSO), with a TOF of 2380 h^−1^. Furthermore, the catalytic activity was improved by adding a base compound, such as NEt_3_, at 90 °C with a TOF of 7330 h^−1^ and TON of 1 100,000 in 150 h. The authors studied the reaction mechanism by ^1^H NMR analysis and suggested that the catalysis entailed the ligand aromatization–dearomatization process. In the first step, protonation (aromatization) of the imine nitrogen occurs, and the formate coordinates to the metal center. Subsequently, a dihydride complex is generated via CO_2_ elimination. Finally, the dihydride complex eliminates H_2_ gas via ligand dearomatization, leading to regeneration of the dearomatized pincer complex (Figure 2).

**Figure 3:**
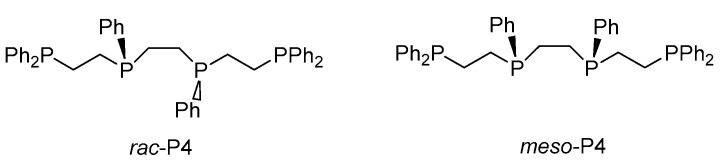
Structures of *rac-* and *meso*-P4.

Gonsalvi et al. developed an in-situ catalyst system for FADH, with a Ru complex (**Ru-3**) in the presence of a linear tetraphos ligand, P4 (1,1,4,7,10,10-hexaphenyl-1,4,7,10-tetraphosphadecane) [15]. Among the two stereoisomers (Figure 3), the *meso* isomer was more effective for Ru catalysis than the *rac*-isomer. The authors investigated the catalyst durability in a long-term reaction, using a pump to feed an FA solution. [RuCl_2_(benzene)]_2_/*meso*-P4 (1/2) was employed (conditions: FA/DMOA (11:10) 5.0 mL, PC 1.0 mL at 60 °C), and the catalyst mixture was recyclable 11 times, with a total TON of 220 000 in 48 h. However, *rac*-P4 could be recycled for only eight consecutive runs when it was used as a ligand under similar reaction conditions, although the mixture showed catalytic activity. No CO was detected in the produced H_2_/CO_2_ gas. This gas can be supplied directly to the H_2_/O_2_ PEM fuel cell to generate electricity. The fuel cell could generate a stable current to drive the fan, owing to the continuous gas supply.

Subsequently, Huang et al. developed a Ru complex (**Ru-4**) with a bisimidazoline ligand for FADH in H_2_O because Li et al. demonstrated that the bisimidazoline ligand of an Ir complex was effective for FADH [16,32]. Although the catalytic activity of the Ru complex was lower than that of the Ir analog, a TOF of 12,000 h^−1^ and TON of 350,000 were achieved with HCOONa at 90 °C. In addition, highly pressurized H_2_/CO_2_ (24 MPa) was produced, without CO contamination. Recently, Milstein et al. obtained a high TON of more than 1.7 million and TOF of 3067 h^−1^, with **Ru-5** bearing a 9H-acridine pincer ligand as the FADH catalyst at 95 °C [17]. The characteristic feature of this catalytic system is that it maintains catalytic activity for two months and has high durability in a neat solvent.

**Figure 4:**
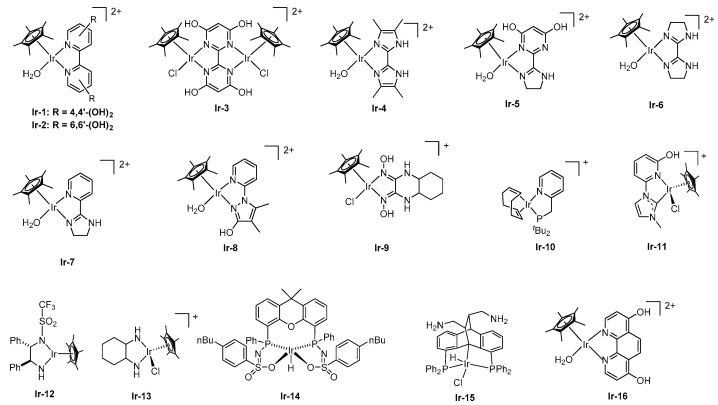
Ir catalysts for FADH.

**Figure 5:**
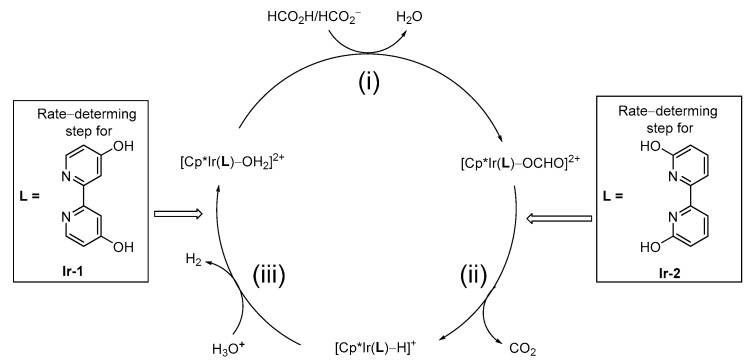
Proposed mechanism and different rate-determining steps of **Ir-1** and **Ir-2** [19].

Various iridium catalysts are also reported to be effective (Figure 4). In 2009, Himeda discovered that Cp*Ir catalysts with N^N-bidentate ligands, such as 2,2′-bipyridine (BPy), catalyzed FADH [18]. This type of catalyst, which is water-soluble, is catalytically active toward aqueous FADH without organic bases, such as amine derivatives. Himeda et al. found that the electron-donating property of the ligand affected the improvement of catalytic performance. For example, Cp*Ir catalysts bearing 4,4′-dihydroxy-bipyridine (**Ir-1**) showed 80-fold higher catalytic activity than the BPy catalyst for FADH. Next, when the OH group on the ligand was moved to the ortho position, interesting findings were obtained from the viewpoint of the reaction mechanism [19,33]. First, the pH dependence of the catalytic activity differed greatly, depending on the substitution position of the OH group. Although the catalytic performances of **Ir-1** and **Ir-2** were almost similar under acidic conditions (pH 1.7), the catalytic activity of **Ir-1** decreased as the pH increased. In contrast, **Ir-2** showed the maximum TOF (5440 h^−1^) at pH 3.5. Subsequently, using deuterated FA (DCOOD) and D_2_O, the rate-determining step differed, depending on the position of the OH substituent because of the kinetic isotope effect (KIE). In FADH, it is speculated that the reaction proceeds through the following three elementary reactions: (i) formation of the formato complex by the reaction of the catalyst precursor and formate ion, (ii) formation of the Ir–H complex by the β-elimination of CO_2_, and (iii) hydrogen production by reaction of the Ir–H complex and a proton. The estimated rate-determining step (RDS) of **Ir-1** was (iii) by KIE experiments, while the considered RDS of **Ir-2** was (ii), owing to the pendant-base effect of the OH groups near the metal (Figure 5). This consideration is supported by the DFT calculations.

Ir catalysts bearing THBPM (THBPM = 2,2′,6,6′-tetrahydroxyl-4,4′-bipyrimidine) (**Ir-3**), which has four OH groups on the ligand, showed high catalytic activity with a TOF of 158,000 h^−1^ and TON of 308,000 in a 1 M aqueous FA/HCO_2_Na (SF) solution at 80 °C [20]. Because **Ir-3** also demonstrated high performance for the hydrogenation of CO_2_ to formate, a reverse reaction of FADH, it was possible to perform the interconversion of CO_2_/FA. It was proved that both hydrogen storage and hydrogen generation reactions could be performed in one batch by performing both cycles twice.

**Figure 6:**
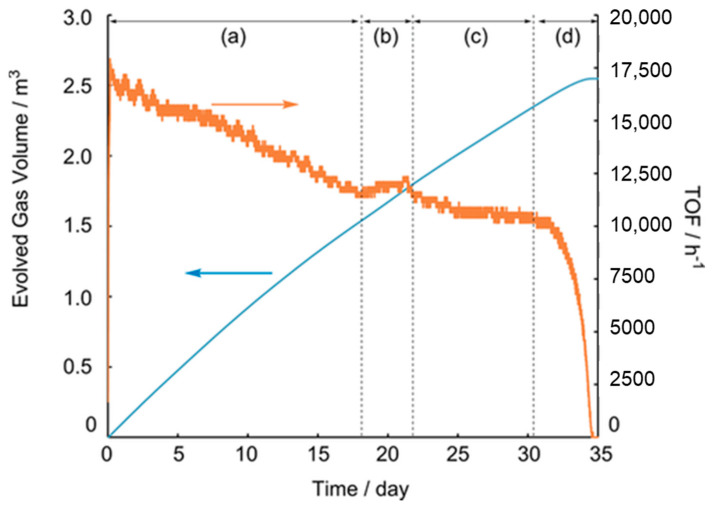
Time courses of volume of released gases (blue line) and rate of gas release (orange line) in FADH, with the continuous addition of FA by a pump. Conditions: [FA]_0_ = 4.0 M, 500 mL, [**Ir-8**]_0_ = 10 μM, [FA]_add_ = 80 wt% (= 20 M), at 70 °C. Rate of FA addition: (**a**) = 0.07 mL/min, 420 h, 35.3 mol, (**b**) = stop, (**c**) = 0.05 mL/min, 250 h, 15 mol, (**d**) = stop, total FA amount = 52.3 mol [24]; © 2022 Wiley.

Subsequently, Himeda et al. reported that an Ir catalyst-bearing a five-membered ring, such as imidazole or imidazoline, could be an effective ligand bone. Tetramethyl biimidazole was an effective ligand framework in the Cp*Ir catalyst (**Ir-4**), which exhibited a TOF of 34,000 h^−1^ [21]. Furthermore, they synthesized an imidazoline-based ligand for the first time, using a combination of dihydroxypyrimidine (**Ir-5**) [22]. **Ir-5** exhibited a high TOF of 322,000 h^−1^ with a TON of 68,000, under reflux conditions in 4M FA/SF. Furthermore, a Cp*Ir catalyst-bearing an azole-based ligand with hydroxy pyrimidine reached a high TON in 6 M FA at 60 °C (TON = 2,000,000). Independently, Li et al. developed a novel Ir complex, bearing a bisimidazoline ligand (**Ir-6**), which showed the highest catalytic activity in water, with a TOF of 487,500 h^−1^ [32]. The pyridyl-imidazoline ligand was also effective for FADH [23]. Although **Ir-7** demonstrated lower catalytic activity than **Ir-6**, its robustness was remarkably improved, due to the combination of a robust pyridyl moiety and active imidazoline moiety. Furthermore, FADH catalyzed by **Ir-7** was performed in 1 L of a 10 M FA solution at 50 °C to produce a large volume of H_2_. After the consumption of approximately 80% of the loaded FA, an additional 10 mol of FA was added. Finally, the reaction was completed with the generation of 1 m^3^ of gas, without critical deactivation. However, **Ir-7** was deactivated under reflux conditions (conditions: [FA] = 8.0 M, 100 mL, [cat] = 10 μM, at 110 °C). Conversely, **Ir-8** completely consumed FA, even under reflux conditions [24]. Additionally, **Ir-8** maintained its catalytic activity during a long-term reaction (35 d), with the continuous addition of FA using a pump (Figure 6). Finally, a total TON of 10 million was achieved, as well as the evolved gas volume of 2.5 m^3^, which was the highest to be reported. Subsequently, Li et al. employed a glyoxime ligand as a new ligand framework [25]. Using a novel Cp*Ir catalyst (**Ir-9**) in a 10 M FA solution at 90 °C, they achieved a high TON of 3,900,000 and average TOF of 65,000 h^−1^.

Williams et al. reported that FADH was catalyzed by the Ir catalyst **Ir-10**, which bears a pyridyl phosphine ligand converted to the active dimeric species via the elimination of a cyclooctadiene moiety [26]. Remarkably, this catalysis could proceed without any solvent in a neat FA solution. In addition, the neat FA could be utilized without purification for this reaction. In the presence of HCOONa as a cocatalyst (5 mol%), a TON of 12,530 was obtained at 90 °C after 13 h. Furthermore, a pale orange residue, composed of an iridium complex and HCOONa, remained in the reaction vessel after the completion of the reaction, which was reused for the next neat FADH. Therefore, the reaction was restarted by feeding neat FA into the reaction vessel at 90 °C, without other operations to regenerate the catalyst. Notably, repeated experiments resulted in a TON of 2,160,000 and TOF of 3.7 s^−1^. According to the GC measurement, the gas obtained from this reaction consisted only H_2_ and CO_2_ and contained almost no detectable CO. Papish et al. reported NHC as an effective ligand framework in Cp*Ir catalyst (**Ir-11**) for FADH [27]. The NHC moiety in the ligand was expected to form a stable metal–ligand bond, owing to its high electron-donating ability. However, the catalytic performance of the complex did not reach the same level as that of the other Cp*Ir catalysts with an N^N-bidentate ligand. Ikariya et al. investigated the Cp*Ir complex with N-triflyl-1,2-diphenylethylenediamine (**Ir-12**) for FADH at ambient temperature without base additives [28]. Particularly, the isolation of the Ir–H complex, a reaction intermediate generated by the reaction of **Ir-12** with FA, showed that the catalytic activity for FADH was almost similar to that of the original complex. Moreover, the addition of water improved the catalytic activity, leading to a maximum TOF above 6000 h^−1^. A proton relay mechanism, guided by the protic amine ligand and water, was postulated for the effective protonation of the metal hydrides. Laurenczy et al. investigated various non-aromatic diamine-type ligands for Cp* Ir-catalyzed FADH, which demonstrated high catalytic activity in water without any organic amines [29]. In particular, the complex bearing 1,2-diaminocyclohexane (**Ir-13**) achieved the highest performance, with a TOF of 3300 h^−1^ at 90 °C. In general, many effective ligands for Cp*Ir catalysts for FADH are aromatic rings, such as pyridine. Therefore, the aforementioned results obtained for non-aromatic diamine-type ligands were significant for developing new ligands. Reek et al. reported an Ir catalyst (**Ir-14**) with a bisMETAMORPhos ligand that has an internal base functionality [30]. In this catalytic system, introducing an electron-donating substituent into sulfonamide enhanced the catalytic activity (up to a TOF of 3090 h^−1^) at 85 °C. NMR and computational studies suggested that the hydrogen bonding interactions between FA and the ligand contributed to the stabilization of intermediates and transition states, leading to an unusual direct hydride transfer mechanism, instead of the conventional β-hydride elimination for FADH. In 2017, Gelman et al. reported a new bi-functional Ir catalyst, bearing PCP ligand for FADH [31]. **Ir-15** achieved a TON of up to 500,000 and a TOF of 20,000 h^−1^ in DME at 70 °C in the presence of 30 mol% HCOONa. From experimental and mechanistic studies, hydrogen is liberated through intramolecular protonolysis of the Ir–H bond and the formation of a cationic species stabilized by the amine chelation from the amino group in the backbone. Regeneration of the active intermediate was performed via an outer-sphere intramolecular β-H elimination of CO_2_.

In FADH, it is necessary to remove CO_2_ gas from the mixed gas to provide highly purified H_2_ for fuel cell vehicles. Thermodynamically, FADH proceeds, even at high pressures under mild temperatures, owing to entropy changes during the reaction (G° = −32.9 kJ/mol). The pressurization and subsequent FADH into H_2_ avoids the consumption of H_2_ compression energy. For purification, FADH was allowed to proceed in a closed vessel. Kawanami et al. found that **Ir-16** was involved in FADH under evolved H_2_/CO_2_ pressures of up to 123 MPa at 80 °C [34,35,36]. Under the reaction conditions, the generated gas mixture was in the supercritical phase; therefore, it allowed H_2_ purification via a simple reduction of the temperature. Cooling to −51 °C separated the two phases, that is a CO_2_-rich liquid and 85 mol% pure H_2_ gas phase. They concluded that the H_2_ release from FA in this manner could sufficiently feed a fuel cell vehicle that typically stores a high-purity carrier at 70 MPa, thus avoiding the need for a costly high-pressure pump.

## 3. Non-Precious Metal Catalysts for FADH

**Figure 7:**
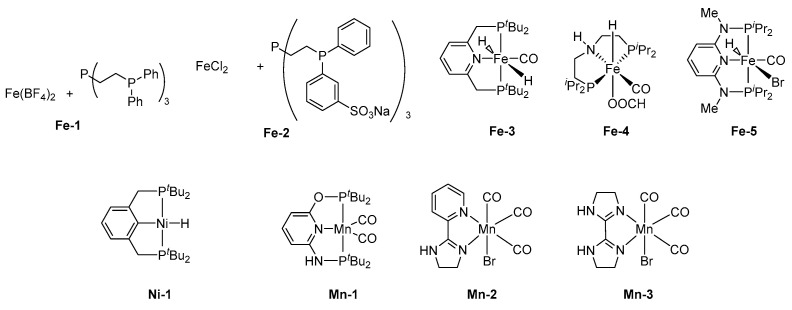
Non-precious metal catalysts for FADH.

Precious metals are often used as the central metal for highly active catalysts. However, the development of inexpensive metal catalysts is essential, due to the possibility of resource depletion. Although several non-precious metal catalysts for FADH have been reported, they exhibit low catalytic activity. However, various non-precious metal catalysts, including iron [37,38,39], nickel, cobalt [40,41], copper [42], and aluminum [43,44], have also been recently reported. The following is a summary of the recent notable achievements (Figure 7, Table 2).

In 2011, an important paper on iron catalysts was reported by Beller et al. [45]. Particularly, Fe(BF_4_)_2_·6H_2_O was employed for the catalytic FADH, in the presence of a tetradentate tris[2-(diphenylphosphino)ethyl]phosphine (tetraphos, PP_3_) ligand with high activity (**Fe-1**). The Fe/PP3 system converted all of the FA to generate H_2_/CO_2_ (1/1) without the CO byproduct (<1 ppm) at 40 °C. A high TOF (9425 h^−1^) was obtained at 80 °C, with a low catalyst loading (0.005 mol%) and Fe/PP3 (1/4). Additionally, the stability of this catalytic system was examined. FA was continuously added to the catalytic solution (Fe/4PP3) via a pump (0.27 ± 0.04 mL min^−1^), and the reaction was observed. Remarkably, a stable TOF (5390 h^−1^) was obtained over 16 h, and the total TON reached 92,000. Subsequently, they reported the results of a detailed investigation of the effect of various ligands, metal salts, solvents, and additives [56]. A decrease in the catalytic activity was observed when water was added. The DFT calculations and spectroscopic investigations (IR, Raman, UV–vis, and XAS) identified the iron η^2^-formate [Fe(η^2^-O_2_CH)(PP3)] as the key active species for the catalytic reaction. However, this active species was deactivated in the presence of chloride ions. Subsequently, Laurenczy et al. investigated an aqueous iron catalyst system for FADH, using FeCl_2_ and water-soluble *m*-trisulfonated-tris[2-(diphenylphosphino)ethyl]phosphine sodium salt as a PP3 derivative (**Fe-2**) at 80 °C [46]. The catalytic system was active for FADH, with a TOF of up to 240 h^−1^. The gases produced were CO-free (<5 ppm). In addition, the catalytic system was recycled four times, without any significant loss of activity.

**Figure 8:**
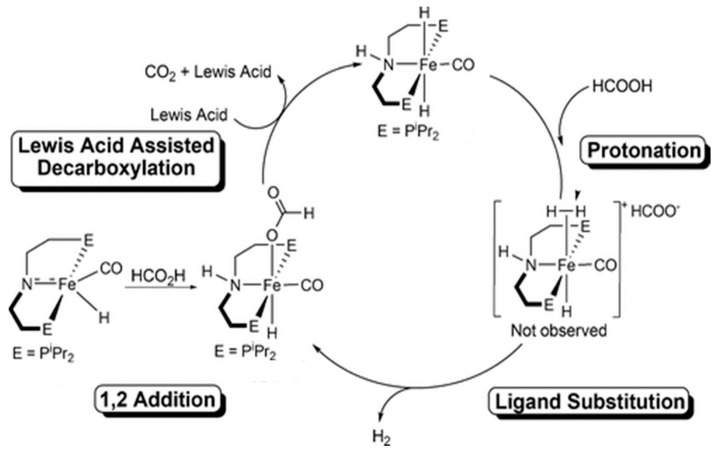
Proposed reaction mechanism for FADH catalyzed by **Fe-4** in the presence of a Lewis acid [48] © ACS 2014.

Catalysts bearing the pincer-type ligands have also been extensively studied for FADH. In 2013, Milstein et al. reported that the Fe PNP-pincer complex (**Fe-3**) trans-[Fe(*t*BuPNP)(H)_2_(CO)] (0.001 mol%) catalyzed FADH in the presence of 50 mol% NEt_3_ in dioxane, with a TON of 100,000 at 40 °C [47]. This catalyst exhibited excellent stability and maintained its catalytic performance for 10 d. Experimental studies revealed that the catalytic process proceeded via protonation of the iron dihydride catalyst, followed by the liberation of dihydrogen and conversion of unsaturated species to the hydrido–formate complex. Regeneration of the iron hydride catalyst was achieved via CO_2_ elimination. Hazari et al. reported that the catalytic activity of a Fe complex (**Fe-4**) bearing a PNP-pincer ligand, [(PNP)Fe(CO)H(COOH)] (PNP = HN[C_2_H_4_(P*i*Pr_2_)]_2_), improved in the presence of a Lewis acid as a cocatalyst [48,57]. FADH was achieved using this iron catalyst (0.0001 mol%), in the presence of LiBF_4_ (10 mol%) in dioxane at 80 °C, with a high TOF of 196,700 h^−1^ and a TON of 1,000,000 in 9.5 h. They proposed that Lewis acids assisted in the decarboxylation of the Fe–formate intermediate to generate the Fe–hydride complex (Figure 8).

Gonsalvi et al. investigated an effective Fe complex (**Fe-5**)-bearing 2,6-diaminopyridine as a PNNNP pincer ligand for FADH. No reactions were observed in the absence of a base [49]. However, adding a base, such as NEt_3_ (50 mol%), improved the catalytic activity, with a TOF of 276 h^−1^ at 60 °C. FADH, using this catalyst in propylene carbonate (PC), with NEt_3_ at 80 °C, yielded a TON of 10,000 and TOF of 2635 h^−1^, with the complete consumption of FA. Further, the authors investigated the role of amines, using NMR experiments, and found that the amines released Br and promoted the regeneration of the reaction intermediate. Gonsalvi et al. also reported that the tetraphos ligand P4 (Figure 3) was effective for iron-catalyzed FADH. This linear tetraphos ligand could have two isomers, *meso*- or *rac*-(**Fe-6**) [50]. The catalytic activity of the *rac*-isomer was higher than that of the *meso* isomer. It was also found that the ratio of Fe to the tetraphos ligand has a significant influence on the catalytic activity, as 1 to 4 showed higher catalytic activity than 1 and 2. FADH could proceed in PC at 60 °C, with a TON of 6061, without any additives such as a base; the reaction was catalyzed by in-situ-generated **Fe-6**, using *rac*-P4 with Fe(BF_4_)_2_·6H_2_O (Fe/Ligand = 1/4).

Nickel can also be used as a catalyst. Enthaler found that a Ni catalyst (**Ni-1**) with a PCP pincer ligand catalyzes FADH [51]. First, in the presence of FA and NEt_3_, NMR experiments confirmed that FA reacts with the hydride moiety in the Ni complex and FA is completely consumed. Subsequently, when FADH was performed for 2 h in PC, using a Ni catalyst with *n*OctNMe_2_ as an additive, a TON of 481 was obtained, and a small amount of CO was produced. In addition, the catalyst could be reused by adding fresh FA after the completion of the reaction. Notably, the reused catalyst has almost similar catalytic activity, as in the first-stage reaction. Inspired by the work of Enthaler et al., Parkin et al. reported on the zerovalent Ni complex Ni(PMe_3_)_4_ (**Ni-2**), which showed catalytic activity toward FADH, with a TON of 80 and TOF of 1.7 h^−1^ [52]. Reaction intermediate was observed by ^1^H NMR experiments, and it was clarified that the starting material reacted with FA to generate a hydride complex, [Ni(PMe_3_)_4_H]+.

Recently, it has been reported that manganese was used as a catalyst for FADH. In 2019, Tondreau et al. found that [(*t*BuPNNOP)Mn(CO)_2_]Br (**Mn-1**) achieved FADH, with a TOF of 8500 h^−1^ and TON of 20,000, in the presence of NEt_3_ and chlorobenzene at 80 °C [53]. The authors also proved that **Mn-1** was robust toward FADH and could be recycled five times. In 2020, Beller et al. showed that imidazoline-based ligand was effective for manganese-catalyzed FADH [54,55]. A **Mn-2**-bearing pyridyl-imidazoline ligand converted FA to generate 14 L of H_2_/CO_2_ mixture gases (TON of 5736) and was stable for more than 3 d in the presence of KOH in H_2_O/triglyme solution. It was found that the catalyst performance of **Mn-2** deteriorated as the pH rose with the consumption of FA. Therefore, the catalytic performance could be maintained by adding a buffer to maintain a neutral pH. Additionally, it was clarified that another Mn catalyst (**Mn-3**), using an imidazoline derivative as a ligand, was effective for FADH, with a TON of 7500 and a small amount of CO contamination (<50 ppm) in DMOA at 95 °C.

## 4. Conclusions

FA is less toxic under ambient conditions and has a flash point well above room temperature, as well as a hydrogen content of 4.4 wt%, making it one of the most promising materials for hydrogen storage. Today, many highly active and robust homogeneous catalysts that selectively decompose FA into H_2_ and CO_2_ have been developed and elucidated in the literature. Particularly, catalysts that exhibit good performance in organic solvents and water have been developed. In addition, there are catalysts whose catalytic activity and durability have been evaluated, implying that studies on practical applications are imminent. Moreover, the development of effective catalysts that exhibits catalytic activity toward the hydrogenation of CO_2_ to FA or formate is progressing, in order to reduce CO_2_ in the atmosphere. Some of the catalysts introduced in this paper currently are also effective for CO_2_ hydrogenation, and it is expected that the transition to a hydrogen society will be accelerated by combining these well.

It was found that precious metals had higher catalytic performance than non-precious metals. However, the research to improve the catalytic performance of non-precious metals remains vital, considering the cost of the catalysts. It is also important to improve the durability of the catalyst for practical use. A system that stably supplies hydrogen at a constant rate, without deactivating during the reaction, is required. In addition, because hydrogen generated from FA contains CO_2_, it is essential to take measures for removing CO_2_. Although Kawanami et al. attempted to increase the pressure in a sealed state, a low temperature was required. In the future, it will be important to increase the purity of hydrogen, with minimum wastage of energy [58].

## Figures and Tables

**Table 1:** Selected precious metal catalysts for FADH.

Catalyst	Temp., °C	Solvent	Additive	TON	TOF, h^−1^	Ref.
**Ru-1**	90	DMF	NEt_3_	706,500	257,000	[13]
**Ru-2**	90	DMSO	NEt_3_	1,100,000	7330	[14]
**Ru-3**	60	PC	DMOA	220,000	-	[15]
**Ru-** **4**	90	H_2_O	HCOONa	350,000	12,000	[16]
**Ru-** **5**	95	-	-	1,700,000	3067	[17]
**Ir-1**	60	H_2_O	-	5000	2400	[18]
**Ir-2**	60	H_2_O	HCOONa	5300	5440	[19]
**Ir-3**	80	H_2_O	HCOONa	308,000	158,000	[20]
**Ir-4**	80	H_2_O	-	10,000	34,000	[21]
**Ir-5**	100	H_2_O	HCOONa	68,000	322,000	[22]
**Ir-6**	90	H_2_O	-	47,000	487,500	[23]
**Ir-7**	50	H_2_O	-	2,000,000	7340	[24]
**Ir-8**	70	H_2_O	-	10,000,000	-	[24]
**Ir-9**	90	H_2_O	-	3,900,000	65,000	[25]
**Ir-10**	90	-	HCOONa	121,000	674,000	[26]
**Ir-11**	60	H_2_O	-	90	31	[27]
**Ir-12**	35	DME	-	2340	6090	[28]
**Ir-13**	90	H_2_O		3300	3300	[29]
**Ir-14**	85	toluene	-	3090	-	[30]
**Ir-15**	60	DME	HCOONa-	500,000	20,000	[31]

**Table 2:** Selected non-precious metal catalysts for FADH.

Catalyst	Temp., °C	Solvent	Additive	TON	TOF, h^−1^	Ref.
**Fe-1**	80	PC	-	92,000	5390	[45]
**Fe-2**	80	H_2_O	-	-	240	[46]
**Fe-3**	40	dioxane	NEt_3_	100,000	-	[47]
**Fe-4**	80	dioxane	LiBF_4_	1,000,000	196,700	[48]
**Fe-5**	80	PC	NEt_3_	10,000	2635	[49]
**Fe-6**	60	PC	-	6061	-	[50]
**Ni-1**	80	PC	OctNMe_2_	626	209	[51]
**Ni-2**	80	C_6_D_6_	-	70	1.7	[52]
**Mn-1**	80	chlorobenzene	NEt_3_	20,000	8500	[53]
**Mn-2**	92.5	H_2_O/triglyme	KOH	5763	-	[54]
**Mn-3**	95	DMOA	-	7500	-	[55]

## Data Availability

All data are included at the manuscript.
